# Residential Exposure to Road and Railway Noise and Risk of Prostate Cancer: A Prospective Cohort Study

**DOI:** 10.1371/journal.pone.0135407

**Published:** 2015-08-25

**Authors:** Nina Roswall, Kirsten T. Eriksen, Dorrit Hjortebjerg, Steen S. Jensen, Kim Overvad, Anne Tjønneland, Ole Raaschou-Nielsen, Mette Sørensen

**Affiliations:** 1 Danish Cancer Society Research Center, Copenhagen, Denmark; 2 Department of Environmental Science, Aarhus University, Roskilde, Denmark; 3 Section for Epidemiology, Department of Public Health, Aarhus University, Aarhus, Denmark; University of Pennsylvania Perelman School of Medicine, UNITED STATES

## Abstract

**Background:**

Few modifiable risk factors for prostate cancer are known. Recently, disruption of the circadian system has been proposed to affect risk, as it entails an inhibited melatonin production, and melatonin has demonstrated beneficial effects on cancer inhibition. This suggests a potential role of traffic noise in prostate cancer.

**Methods:**

Road traffic and railway noise was calculated for all present and historical addresses from 1987–2010 for a cohort of 24,473 middle-aged, Danish men. During follow-up, 1,457 prostate cancer cases were identified. We used Cox Proportional Hazards Models to calculate the association between noise exposure and incident prostate cancer. Incidence Rate Ratios (IRR) were calculated as crude and adjusted for smoking status, education, socioeconomic position, BMI, waist circumference, physical activity, calendar year, and traffic noise from other sources than the one investigated.

**Results:**

There was no association between residential road traffic noise and risk of prostate cancer for any of the three exposure windows: 1, 5 or 10-year mean noise exposure before prostate cancer diagnosis. This result persisted when stratifying cases by aggressiveness. For railway noise, there was no association with overall prostate cancer. There was no statistically significant effect modification by age, education, smoking status, waist circumference or railway noise, on the association between road traffic noise and prostate cancer, although there seemed to be a suggestion of an association among never smokers (IRR: 1.16; 95% CI: 1.00–1.36).

**Conclusion:**

The present study does not support an overall association between either railway or road traffic noise and overall prostate cancer.

## Introduction

Prostate cancer is the second most common non-skin cancer in men worldwide [1], and the incidence is rising in most countries [2, 3]. The strongest known risk-factors for disease include a range of non-modifiable factors, such as age, race, and family history [3, 4], whereas evidence is less strong for modifiable lifestyle-factors [5]. Thus, further research into these is required in order to advance disease prevention.

A recent study found exposure to traffic noise to be associated with higher risk for breast cancer [6]. Road traffic noise is a main source of noise in larger cities, and nocturnal traffic noise has been shown to cause sleep disturbance [7–9]. Studies on sleep disruption and prostate cancer are few: One cohort study found a significant inverse association between sleep duration and prostate cancer risk [10], and a study on fatal prostate cancer found that short sleep duration (<7 hours/night) was associated with a higher risk of death during the first eight years of follow-up, but found no association with insomnia frequency [11]. These studies may, however, be affected by reverse causality, as an undetected cancer could affect sleep quality/duration; thus being the cause rather than the effect [12]. In addition, some epidemiological studies have reported that night shift work, representing a massive disturbance of sleep and the circadian rhythm, is associated with an increased risk for prostate cancer [13–15].

A potential mechanism through which sleep disruption may affect prostate cancer risk is suppression of melatonin [16]. This is supported by a recent, Icelandic case-cohort study, which found that men who reported sleeping problems had lower levels of first morning-void urinary 6-sulfatoxymelatonin (aMT6s)–the primary urinary melatonin metabolite- compared to men who reported no sleeping problems. Furthermore, men with low aMT6s concentration had a statistically significant higher risk for advanced prostate cancer [17]. Other studies have also indicated that sleep disruption suppresses melatonin production, and may thus affect cancer risk [16, 18]. Melatonin has been found to inhibit prostate tumor growth in vitro as well as in vivo [19, 20]. Suggested anti-carcinogenic mechanisms of melatonin includes inhibition of tumor initiation by preventing DNA damage and promoting DNA repair [21], and tumor promotion and progression through effects on anti-angiogenesis, induction of apoptosis, reduction of cell proliferation and metabolic activity of cancer cells [16, 21–24]. This suggest a role of melatonin in the etiology of prostate cancer, and it is supported by human studies showing a negative association between tumor size and melatonin levels in prostate cancer patients [25], and a cross-sectional study finding lower serum melatonin levels in men with prostate cancer compared to men with benign prostatic hyperplasia [26].

Based on these findings, we hypothesize, that residential traffic noise exposure may be associated with prostate cancer risk. Thus, the aim of the present study is to investigate the association between residential exposure to road traffic and railway noise and the risk of incident prostate cancer in a population-based cohort of 24,473 middle-aged, Danish men. Furthermore, we examine potential effect modification by age, education, smoking, waist circumference and railway noise.

## Materials and Methods

### Study population

From December 1, 1993, through May 31, 1997, 27,178 men aged 50–65 years, born in Denmark, with no previous cancer diagnosis, and living in Greater Copenhagen or Aarhus were enrolled in the prospective, Danish Diet, Cancer and Health (DCH) cohort [27]. At enrolment, the participants completed a self-administered, interviewer-checked food frequency questionnaire and a questionnaire covering lifestyle habits including information on smoking habits, physical activity, social factors, and health status. Height, weight, and other anthropometric measurements were measured by trained staff members according to standardized protocols.

We used the nationwide Danish Cancer Registry, containing accurate and virtually complete data on cancer incidence in Denmark, to identify all cases of prostate cancer in the cohort from baseline to December 31, 2010 [28]. Definition of prostate cancer was based on the 10th Revision of the International Classification of Diseases: C61. To further categorize prostate cancer we applied an algorithm based on modifications of the D’Amico [29] and the TNM classifications to divide cases into either aggressive or non-aggressive tumors. For cases diagnosed up until December 31, 2008, we obtained the following data from a thorough review of medical records: Gleason score, PSA test results at diagnosis, size and extent of the tumor (T), degree of spread to regional lymph nodes (N) and the presence of metastasis (M). Cases fulfilling one or more of the following criteria’s were classified as aggressive: Gleason score ≥7, PSA >15, T-stage ≥3, N-stage ≥1, or M-stage ≥1. For cases who did not have complete information available, the records were re-examined by a medical doctor and classified according to aggressiveness if feasible.

The study was approved by the local ethical committees of Copenhagen and Frederiksberg Municipalities (in Danish: "*Den Videnskabsetiske komite for Københavns og Frederiksberg Kommuner*") Approval no.: (KF) 01-345/93. All participants provided written informed consent, and the study was conducted according to the Helsinki Declaration.

### Exposure assessment

Residential address history for all male cohort members between July 1^st^, 1987 and censoring was collected using the Danish civil registration system [30]. Road traffic noise exposure was calculated for the years 1990, 1995, 2000, 2005 and 2010 for all present and historical addresses using SoundPLAN; a software implementing the joint Nordic prediction method for road traffic noise [31]. Using this method, the equivalent noise level can been calculated for each address in a position on the most exposed facade of the actual building and in each of the time periods: day (07–19), evening (19–22) and night (22–07), when a series of traffic parameters and topographical parameters are known. These input variables were: point for noise estimation, corresponding to geographical coordinate and height (floor) for each residential address, road links with information on annual average daily traffic, vehicle distribution (light and heavy in each of the three time periods), travel speed (same for the three time periods) and road type; and building polygons for all Danish buildings provided by the Danish Geodata Agency. We obtained traffic counts for all Danish roads with more than 1,000 vehicles per day from a national road and traffic database [32]. This database is based on different traffic data sources, mainly collection of traffic data from the 140 Danish municipalities with most residents, covering 97.5% of the addresses included in the present study. Included roads typically have more than 1,000 vehicles per day and are based on traffic counts as well as estimated/modelled numbers. Traffic data represents the period from 1995–1998. When calculating road traffic noise for 2000, 2005 and 2010 we included new roads constructed in the relevant period and extrapolated the results from 1995 according to a traffic growth of approximately 10% per 5 years.

We assumed that the terrain was flat, which is a reasonable assumption in Denmark, and that urban areas, roads, and areas with water were hard surfaces, whereas all other areas were acoustically porous. No information was available on noise barriers or road surfaces. All modelled values of road traffic noise below 40 dB were set to 40 dB as this was considered as the lower limit of road traffic noise.

Railway traffic noise exposure was calculated for the years 1990, 1995, 2000, 2005 and 2010 for all present and historical addresses using SoundPLAN, with implementation of NORD2000; a Nordic calculation method for prediction of noise propagation for railway traffic noise. The input variables for the noise model were point for noise estimation (geographical coordinate and height), railway links with information on annual average daily train lengths, train types, travel speed (information obtained from BaneDanmark, which is operating and developing the Danish state railway network); and building polygons for all Danish buildings. The daily train lengths are given for 1997 and 2012. All noise barriers along the railway are included in the model. Railway traffic noise was expressed as L_den_ at the most exposed facade of the dwelling.

The noise impact from all Danish airports and airfields was determined from information about noise zones (5 dB categories) obtained from local authorities. The curves for aircraft noise were transformed into digital maps and linked to each address by geocodes.

Ambient air pollution (NO_x_) was calculated for each year at each address using AirGIS, which calculates a sum of local, urban and regional contributions [33]. Input data included traffic data as described above, emission factors, street and building geometry, and meteorological data [32, 34]. We used NO_x_ as a proxy of traffic-related air pollution as it has been shown to correlate closely with particulate matter, including ultrafine particles and PM_10_ in Danish streets [35].

### Statistical analyses

Cox proportional hazard models were used for statistical analyses. Age was used as underlying time scale, ensuring comparison of individuals of same age. We used left truncation at age at July 1, 1997 (to ensure at least 10 years of exposure history), and right censoring at age of cancer diagnosis (except non-melanoma skin cancer), death, emigration, or December 31, 2010, whichever came first.

Exposure to road traffic noise was modeled as time-weighted averages for the 1, 5, and 10 years preceding prostate cancer diagnosis, respectively, taking all present and historical addresses in that period into account. Exposure to railway noise was modeled as average yearly exposure at the current residence. These exposure windows were entered as time-dependent variables; thus, exposure was estimated for all cohort members who were at risk of diagnosis at exactly the same age as each case at diagnosis.

We estimated incidence rate ratios (IRRs) for the association between road traffic and railway noise and prostate cancer crude (Model 1) and adjusted for the following *a priori* defined potential confounders: educational level (<8 years; 8–10 years; >10 years); area level socioeconomic position (SEP) of baseline municipalities or districts for Copenhagen municipality (in total 10 districts) in three groups (low, medium and high) based on municipality/district information on education; work market affiliation; income; smoking status (never, former and current); smoking duration (years), body mass index (BMI, kg/m^2^, continuous); waist circumference (cm, continuous); physical activity (metabolic equivalent (MET) score, continuous); calendar-year (time-dependent in 5 years intervals); and airport noise (≤45 dB (yes/no)) (Model 2). In addition, road traffic noise and railway noise were mutually adjusted. Lastly, we further adjusted for air pollution (NO_x_, μg/m^3^) calculated as 1-, 5- and 10-years’ time-weighted mean preceding diagnosis, such that in each analysis the exposure calculation for NO_x_ were similar to the exposure calculation for road traffic noise (Model 3).

We calculated separate IRRs for aggressive and non-aggressive prostate cancer, respectively, treating the different types of prostate cancer as competing causes of failure.

Potential modification of the association between road traffic noise and prostate cancer by baseline characteristics as well as age and railway noise exposure at diagnosis were evaluated by introducing interaction terms into the model, and were tested by the Wald test.

The assumption of linearity of L_den_ and covariates was evaluated visually and by formal testing with linear spline models. We found no significant deviation from linearity. For statistical analyses we used the procedure PHREG in SAS version 9.3 (SAS Institute, Cary, North Carolina, USA). The graphical presentation of a functional form of an association between L_den_ and prostate cancer was produced using restricted cubic spline in R, version 3.0.2.

## Results

Among the 27,178 men in the cohort, we excluded 234 with cancer before enrolment, 1 with unknown month of cancer diagnosis, 1,908 with incomplete address history, 179 with missing covariates, and 383 censored before July 1997, leaving a study cohort of 24,473 men. During a mean follow-up time of 13.4 years, prostate cancer was diagnosed in 1,457 of the men.

**Fig 1:**
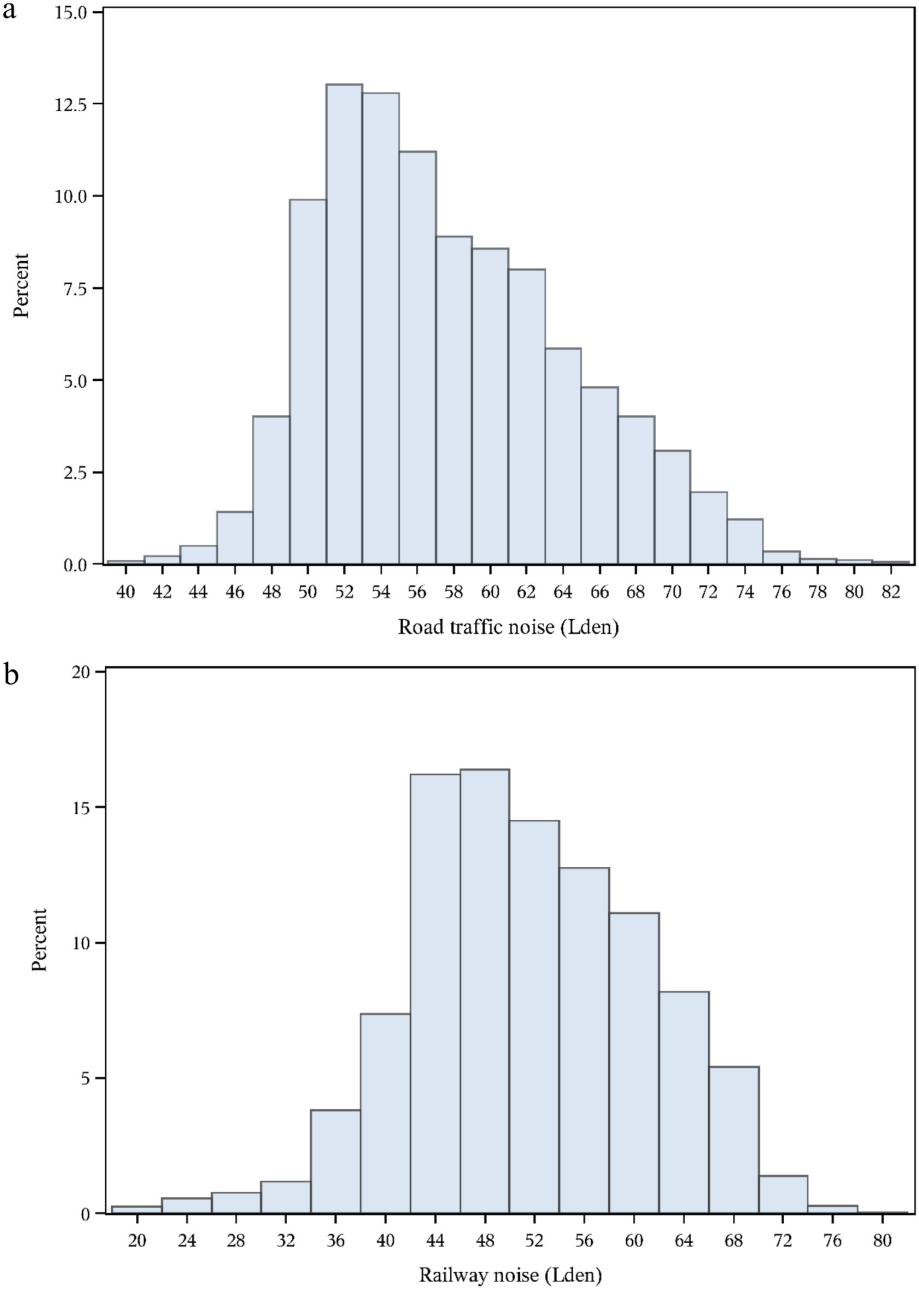
Distribution of residential exposure to road traffic noise (a) and railway noise among exposed participants (b) at the time of enrolment into the cohort.

Distributions of baseline characteristics of prostate cancer cases and non-cases are shown in Table 1. Prostate cancer cases were older at enrolment, had a higher level of education, were less likely to be smokers and were more physically active compared to non-cases. The distribution of road traffic noise (all exposed) and railway noise among exposed participants are shown in Fig 1. There was a very high correlation between exposure to L_den_ road and L_night_ road: R_s_ = 0.999. The correlation between L_den_ road and L_den_ railway among the participants exposed to railway noise (18% at start of follow-up) was weak and insignificant: Rs = 0.02, P = 0.13.

**Table 1:** Baseline characteristics of the 24,473 male participants in the Diet, Cancer and Health cohort by prostate cancer status.

Baseline characteristics	Non-cases (n = 23,016)	Prostate cancer cases (n = 1,457)
**Age (years)**	55.9 (50.7–64.2)	58.1 (51.0–64.7)
**Years of school attendance (%)**		
< 8	35.2	34.4
8–10	41.8	40.5
˃ 10	23.0	25.1
**Area level SEP** ^**1**^ **(%)**		
Low	14.5	13.6
Medium	64.5	63.6
High	21.0	22.9
**Smoking status (%)**		
Never	25.9	27.7
Former	34.6	37.1
Present	39.5	35.3
**BMI (kg/m** ^**2**^ **)**	26.2 (21.5–33.0)	26.1 (21.5–32.2)
**Waist circumference**	95.0 (81.0–114)	95.0 (82.0–113)
**Physical activity (MET score)**	54.0 (16.5–150)	55.5 (16.0–163)
**L**_**den**_ **road traffic (dB)**	56.4 (48.5–70.2)	56.5 (48.9–70.2)
**Railway noise (% exposed)**	18.0	18.2
**Air pollution (NO**_**x**_ **)**	20.8 (14.4–85.9)	20.8 (14.4–91.3)

Numbers are medians (5–95% percentiles) unless otherwise specified

^1^Socioeconomic position of municipalities based on municipality information on education, work market affiliation and income

**Fig 2:**
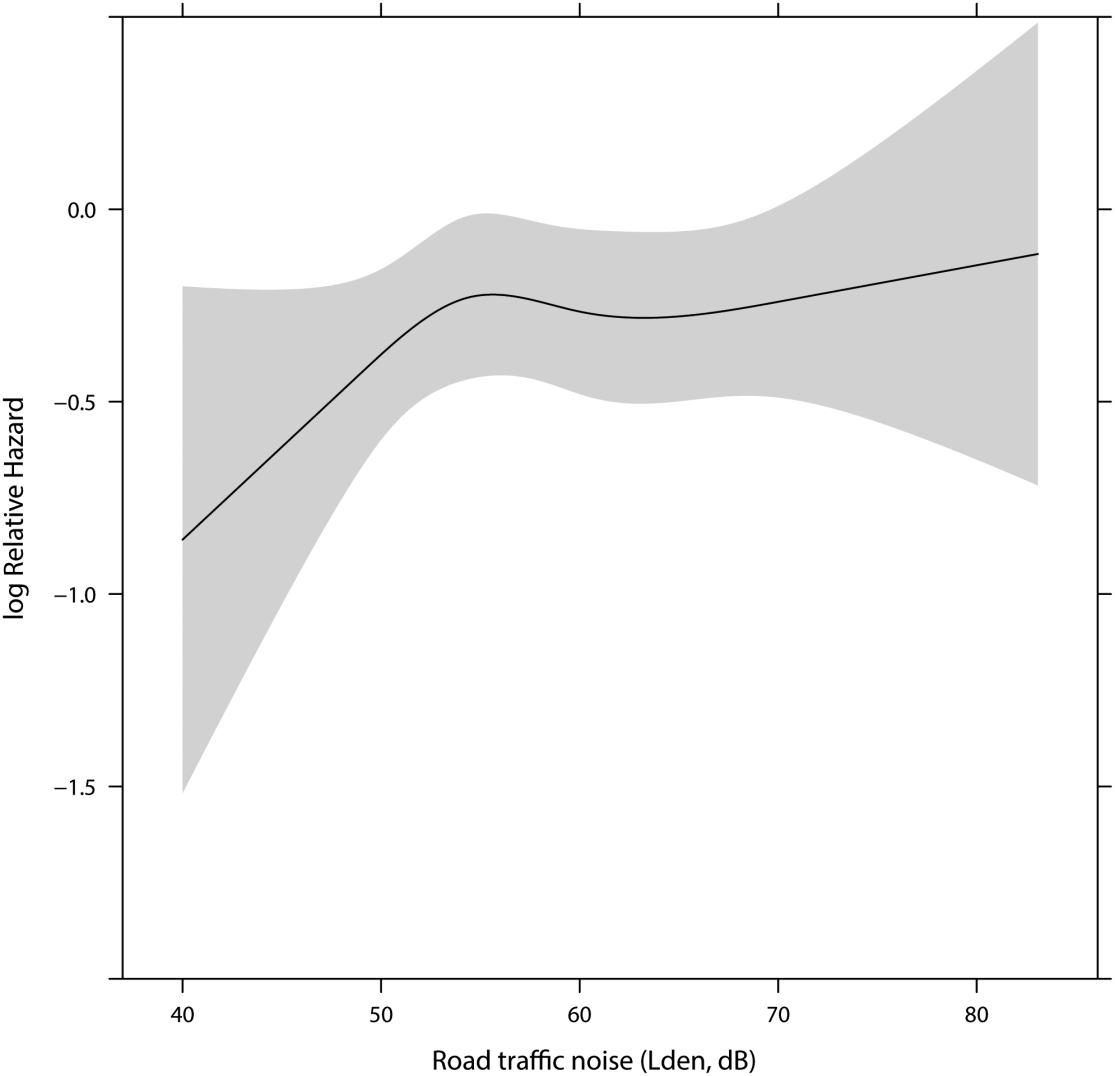
Association between residential exposure to road traffic noise (L_den_) and risk for prostate cancer adjusted for smoking status, years of school attendance, area level socioeconomic position, BMI, waist circumference, physical activity, calendar-year and railway noise and airport noise.

We found no associations between residential road traffic noise and risk of prostate cancer for any of the three exposure windows: 1, 5 or 10-year mean noise exposure before prostate cancer diagnosis (Table 2, Fig 2). Also, there were no associations between road traffic noise and subtypes of aggressiveness (information on aggressiveness was available until 2008, thus, lacking the 2 last years of the follow-up period).

**Table 2:** Incidence rate ratio (IRRs) of prostate cancer per 10 dB higher level of road traffic noise exposure based on 24,473 cohort participants.

Exposure to road traffic noise, L_den_ (per 10 dB)	N cases	Model 1, IRR (95% CI)^1^	Model 2, IRR (95% CI)^2^	Model 3, IRR (95% CI)^3^
**All prostate cancer**	1,457			
L_den_ 1-year preceding diagnosis		1.01 (0.93–1.09)	1.03 (0.95–1.11)	1.06 (0.96–1.17)
L_den_ 5-year preceding diagnosis		1.00 (0.92–1.08)	1.02 (0.94–1.10)	1.04 (0.94–1.15)
L_den_ 10-year preceding diagnosis		1.00 (0.93–1.09)	1.03 (0.95–1.11)	1.04 (0.94–1.15)
**Aggressive prostate cancer** ^4^	777			
L_den_ 1-year preceding diagnosis		1.03 (0.93–1.14)	1.04 (0.94–1.16)	1.13 (0.99–1.29)
L_den_ 5-year preceding diagnosis		1.00 (0.90–1.12)	1.02 (0.91–1.13)	1.10 (0.96–1.26)
L_den_ 10-year preceding diagnosis		1.01 (0.90–1.12)	1.02 (0.91–1.14)	1.10 (0.96–1.27)
**Non-aggressive prostate cancer** ^4^	303			
L_den_ 1-year preceding diagnosis		0.97 (0.81–1.14)	0.99 (0.84–1.18)	0.97 (0.78–1.21)
L_den_ 5-year preceding diagnosis		0.96 (0.81–1.14)	1.00 (0.84–1.19)	0.96 (0.77–1.20)
L_den_ 10-year preceding diagnosis		0.94 (0.79–1.12)	0.98 (0.82–1.17)	0.91 (0.72–1.13)

^1^ Model 1: Crude model

^2^ Model 2: Adjusted for smoking status, smoking duration, years of school attendance, area level socioeconomic position, BMI, waist circumference, physical activity, calendar-year, and railway and airport noise

^3^ Model 3: Model 2 with further adjustment for air pollution (NO_x_)

^4^ Information on aggressiveness was available from enrolment until 2008

We found no association between exposure to railway noise and risk of overall prostate cancer (Table 3). For aggressive prostate cancer, a 10 dB higher railway noise seemed to be associated with a 20% higher risk (95% CI: 1.01–1.43), whereas for non-aggressive prostate cancer railway noise seemed to be associated with a reduction in risk (IRR: 0.80; 95% CI: 0.62–1.03). However, for both types of aggressiveness there seemed to be no clear exposure-response relationship in the categorical analyses: for the aggressive type the IRR’s in the two exposure groups (]0–55 and ≥ 55 dB) were, respectively, lower and higher than the reference group of unexposed, and for the non-aggressive type, exposures above 55 dB were associated with IRR similar to the large reference group of unexposed participants, whereas higher IRR was observed in the lowest exposure category (]0–55).

**Table 3:** Incidence rate ratio (IRRs) of prostate cancer according to railway noise exposure.

Exposure to railway noise, L_den_	N cases	Crude IRR (95% CI)	Adjusted IRR (95% CI)^2^
**All prostate cancer**			
Not exposed	1171	1.00	1.00
< 55 dB	191	0.93 (0.80–1.09)	0.92 (0.79–1.07)
55 dB	95	1.06 (0.86–1.30)	1.07 (0.87–1.32)
*Linear (per 10 dB)* ^*1*^	*286*	*1*.*00 (0*.*88–1*.*14)*	*1*.*02 (0*.*90–1*.*16)*
**Aggressive prostate cancer** ^**3**^			
Not exposed	626	1.00	1.00
< 55 dB	94	0.86 (0.69–1.07)	0.85 (0.69–1.06)
≥ 55 dB	57	1.19 (0.69–1.56)	1.20 (0.91–1.57)
*Linear (per 10 dB)* ^*1*^	*151*	*1*.*18 (0*.*99–1*.*42)*	*1*.*20 (1*.*01–1*.*43)*
**Non-aggressive prostate cancer** ^**3**^			
Not exposed	235	1.00	1.00
< 55 dB	50	1.26 (0.93–1.71)	1.28 (0.94–1.73)
≥ 55 dB	18	1.00 (0.62–1.61)	1.05 (0.65–1.69)
*Linear (per 10 dB)* ^*1*^	*68*	*0*.*78 (0*.*60–1*.*00)*	*0*.*80 (0*.*62–1*.*03)*

^1^ Among the exposed population

^2^ Adjusted for smoking status, smoking duration, years of school attendance, area level socioeconomic position, BMI, waist circumference, physical activity and calendar-year, road traffic noise (10-year mean preceding diagnosis) and airport noise.

^3^ Information on aggressiveness was available from enrolment until 2008

We found no statistically significant effect modification by age, railway noise at diagnosis, and baseline characteristic (Table 4). There was, however, a tendency of a stronger association between road traffic noise and prostate cancer among never smokers (IRR: 1.16; 95% CI: 1.00–1.36) as compared with present (IRR: 0.96; 95% CI: 0.84–1.09) and former (IRR: 1.00; 95% CI: 0.88–1.14) smokers.

**Table 4:** Modification of the association between 10-year mean of road traffic noise (per 10 dB) and risk for prostate cancer by different baseline characteristics and age and railway noise at diagnosis.

Covariates	N cases	Adjusted IRR (95% CI)^1^	*P* interaction
Age (years)			0.62
< 68	722	1.05 (0.93–1.17)	
≥ 68	735	1.00 (0.90–1.13)	
Years of school attendance			0.93
< 8	501	1.04 (0.91–1.19)	
8–10	590	1.02 (0.90–1.15)	
≥ 10	366	1.02 (0.86–1.19)	
Smoking status			0.14
Never	403	1.16 (1.00–1.36)	
Former	540	1.00 (0.88–1.15)	
Present	514	0.95 (0.83–1.09)	
Waist circumference			0.58
< 95 cm	699	1.00 (0.89–1.13)	
≥ 95 cm	758	1.05 (0.94–1.17)	
Railway noise (L_den_)			0.61
Yes	285	1.07 (0.89–1.29)	
No	1172	1.02 (0.89–1.11)	

^1^ Adjusted for smoking status, smoking duration, years of school attendance, area level socioeconomic position, BMI, waist circumference, physical activity, calendar-year and railway and airport noise.

## Discussion

The present study showed no association between road traffic noise and prostate cancer, regardless of tumor aggressiveness and exposure window (1, 5, or 10 years before diagnosis). Similar for railway noise exposure, there was no association with overall prostate cancer, and no clear tendencies with regard to prostate cancer aggressiveness. Finally, we saw no statistically significant effect modification by age, education, smoking status, waist circumference or railway noise, although there seemed to be a suggestion of an association among never smokers.

The study strengths include the prospective design, access to residential address histories, and complete follow-up, with diagnosis of prostate cancer and aggressiveness using nationwide registers. However, some limitations also need to be considered. The study population lived mainly in urban areas and are, thus, not representative for the Danish population. Also, only 36% of the people invited, enrolled into the cohort, and they were found to have higher socioeconomic position than non-participants [27]. Also, some of the sub-analyses, in particular those on railway noise, are based on a relatively modest number of cases. Although the Nordic prediction method has been used for many years, calculation of noise is inevitably associated with some degree of uncertainty. One reason could be inaccurate input data; e.g. lack of information on noise barriers and road surface in the model, which may result in exposure misclassification. As the noise model does not distinguish between cases and non-cases among cohort members, such misclassification is believed to be non-differential, and, in most situations, this would influence the relative risk estimate towards the neutral value. We lacked information on bedroom location, window opening habits, noise from neighbors, or hearing impairment, all of which might influence exposure to noise. Studies on traffic noise and cardiovascular diseases have found that the association with road traffic noise is stronger when these factors are considered [36], suggesting that an effect of noise might be underestimated in the present study. Danish levels of road traffic noise increased only slightly during the study period (approximately 0.4 dB per five years). However, as we adjusted analyses for calendar-year, these modest temporal increases in exposure are not expected to have affected the results. In addition, the method used for classification of prostate cancer aggressiveness was based on a modification of the D’Amico classification, which may have introduced some misclassification.

We have not identified any previous studies investigating the association between residential traffic noise exposure and prostate cancer, and the results of the present study thus require reproduction in other cohorts. However, if the effect of traffic noise operates primarily through sleep disturbance, as several studies have suggested [8, 9, 37], the results of the present study do not support previous studies showing a direct association between sleep disruption and prostate cancer [10, 11, 17], and between night-shift work and prostate cancer [13–15]. However, in the present cohort, we lacked information on actual sleep disturbance as well as on potential mediating factors such as bedroom orientation and window isolation. Residential traffic noise exposure may, thus, not necessarily be a good proxy for sleep disturbance. Another explanation for the null-results of the present study could be that the effect of traffic noise on sleep in the present study is too low to influence the risk for prostate cancer. Similarly, the divergent results between the present study and those on shift work may be explained by the fact that shift work exerts a much larger effect on the circadian system than traffic noise. However, at present little is known on the mechanisms through which shift work affects prostate cancer risk; therefore, a direct comparison with the present results may be difficult, and further research in the field is required, before firm conclusions can be made [17].

Our study indicated that among never smokers there might be an association between exposure to road traffic noise and increased risk for prostate cancer. However, tobacco smoking is not a strong risk factor for prostate cancer, e.g. recent meta-analyses found that current smokers had no increased risk for incident prostate cancer, whereas for fatal prostate cancer the meta-analyses indicated a positive association [38, 39]. Also, adjustment for smoking status and duration in the present study only resulted in minor changes in IRR’s which together with the insignificant test for effect modification indicates that this is a chance finding.

We studied associations between traffic noise and prostate cancer aggressiveness, to investigate if traffic noise might influence the progression of prostate cancer. For road traffic noise we found no marked changes in risk estimates according to aggressiveness, whereas for railway noise stratification by aggressiveness suggested a higher risk of aggressive prostate cancer and a lower risk of non-aggressive prostate cancer. However, this did not seem to follow any exposure-response pattern when comparing with the unexposed participants. Also, this finding cannot be reasonably explained within the above framework or the existing knowledge on the different etiology of the two tumor types, and it may thus be a chance finding due to multiple testing and small numbers. This is supported by the fact that we do not see a similar dual effect of road traffic noise, which is usually classified as more annoying and has been found to cause more awakenings, than train noise [37].

In conclusion, the present study in a cohort of middle-aged, Danish men does not support an overall association between residential traffic noise exposure and risk of prostate cancer. As this is the first study of its kind, we encourage reproduction in other cohorts.
